# Tangent-based volumetric modulated arc therapy for advanced left breast cancer

**DOI:** 10.1186/s13014-018-1167-y

**Published:** 2018-11-28

**Authors:** Pei-Chieh Yu, Ching-Jung Wu, Hsin-Hua Nien, Louis Tak Lui, Suzun Shaw, Yu-Lun Tsai

**Affiliations:** 1 Department of Radiation Oncology, Cathay General Hospital, Taipei, Taiwan; 2 School of Medicine, China Medical University, Taichung, Taiwan; 3 Department of Radiation Oncology, National Defense Medical Center, Taipei, Taiwan; 4 Department of Biomedical Engineering, I-Shou University, Kaohsiung, Taiwan; 5 School of Medicine, Fu Jen Catholic University, Taipei, Taiwan; 6 Oncology Treatment Center, Sijhih Cathay General Hospital, New Taipei, Taiwan

**Keywords:** Tangent-based volumetric modulated arc therapy, Breast cancer, Radiation dose

## Abstract

**Purpose:**

To introduce the benefits of tangent-based volumetric modulated arc therapy (TVMAT), an innovative radiotherapy planning technique, compared with traditional volumetric modulated arc therapy (VMAT) for advanced left breast cancer needing nodal irradiation.

**Materials and methods:**

Twenty-three patients with advanced left breast cancer who had received modified radical mastectomy (MRM) and needed adjuvant radiotherapy including nodal irradiation were assessed. Among 23 radiotherapy treatment plans, 17 plans were designed by using TVMAT technique and 6 plans were designed by using traditional VMAT. The main difference of TVMAT from VMAT was that the area of avoidance sector within specific degrees of angle that had no monitor unit (MU) delivery was used in the arc planning, including a total of 5 sectors in 5 partial arcs. The dosimetries of planning target volume (PTV), right breast, bilateral lungs, and heart between TVMAT and VMAT were compared.

**Results:**

The conformity index (CI) and homogeneity index (HI) of PTV between two groups were statistically equivalent (CI: 0.98 ± 0.02 and 0.98 ± 0.03, *P* = 0.431; HI: 0.12 ± 0.03 and 0.11 ± 0.05, *P* = 0.177), which indicated that the treatment efficacy of the plans regarding TVMAT was compatible with VMAT. However, all neighboring organs at risk (OAR) showed a great percentage of reduction in mean doses (right breast: 53.1%, right lung: 37.7%, left lung: 8.8%, heart: 21.2%) and low dose parameters (V10: right breast: 72.3%, right lung: 86.1%, left lung: 12.5%, heart: 25.1%; V5: right breast: 56.5%, right lung: 28.3%, left lung: 12.7%, heart: 18.2%) by using TVMAT.

**Conclusion:**

TVMAT greatly decreases the radiation doses delivered to the OAR with maintained therapeutic efficacy. It is highly recommended for treating breast cancer, especially for difficult cases with left side disease needing nodal irradiation.

## Introduction

Breast cancer is one of the most commonly diagnosed malignancies globally. The estimated percentage of breast cancers diagnosed with lymph node involvement is around 30% [1]. Nowadays, the general principle of treating breast cancers with nodal disease is to irradiate adjacent lymph node regions along with the primary site after surgery. Including nodal irradiation in the treatment plans can improve survival and reduce mortality from breast cancer [2–4].

When intending to treat lymph node regions, treatment planning is a challenge because the geometry of treatment volumes is much more complicated than breast irradiation alone. Planning is harder for left side diseases, considering the risk of ischemic heart disease after radiotherapy for breast cancer increases linearly with the mean dose to the heart by 7.4% per gray (Gy) with no apparent threshold [5]. Among planning techniques, VMAT allows for a reduction of the maximum doses to OAR, especially to the heart, while retaining target homogeneity and coverage [6]. Otherwise, it reduces the number of MU by 30% and the treatment time by 55% compared with conventional intensity-modulated radiation therapy (IMRT) [7].

Although VMAT planning has the best intermediate-high dose OAR sparing, it compromises the mean and low doses received by the OAR, and it comes also at the expense of more doses to the contralateral lung and breast [8]. This trade-off even presents at the application of deep inspiration breath-hold (DIBH), which shows that a significant increase in the low dose to the heart as well as low and mean doses to the contralateral lung and breast still exists in VMAT [9]. This drives the motivation to explore a better planning technique to improve the disadvantages of VMAT. The study focuses on this issue and introduces TVMAT, an innovative planning technique based on the modifications of VMAT, with the planning objectives, design details, and dosimetry comparisons with VMAT, for treating advanced left breast cancer needing nodal irradiation.

## Materials and methods

### Patient and target volume delineation

**Fig. 1:**
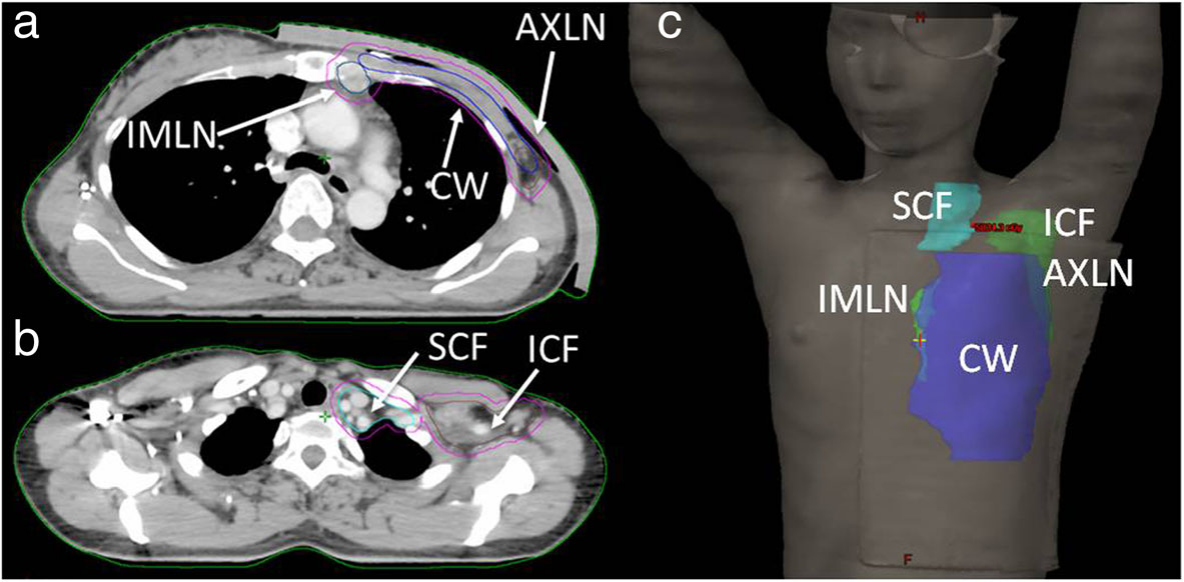
Target volume delineation of advanced left breast cancer. The CTV included **a** chest wall (CW, blue line), axillary lymph node region (AXLN, brown line), and internal mammary lymph node region (IMLN, dark blue line) and **b** supraclavicular fossa (SCF, cyan line) and infraclavicular fossa (ICF, brown line). The PTV (magenta line) was generated by expanding 0.5 cm from the CTV in all directions. **c** overview of target volume delineation

A total of 23 patients with advanced left breast cancer who had undergone MRM and needed adjuvant radiotherapy including nodal irradiation were assessed. Among them, 17 patients received radiotherapy designed by using TVMAT, and the other 6 plans were designed by using traditional VMAT. During computed tomography (CT) simulation, all patients were lying in the supine position with their arms over their heads and immobilized with an extended wing board with T-bar handgrip immobilization devices (CIVCO Medical Solutions, IA, USA). CT images were acquired using the Discovery CT590 RT 16-slice scanner (GE Healthcare, WI, USA) with a slice thickness of 2.5 mm. The scanning range stretched from the second vertebral body of the cervical spine to the forth vertebral body of the lumbar spine. Regarding target volume delineation, Fig. 1 illustrates the locations and contours with respect to different target volumes. The clinical target volume (CTV) included the left chest wall (CW), axillary lymph node region (AXLN), internal mammary lymph node region (IMLN), supraclavicular fossa (SCF), and infraclavicular fossa (ICF). The PTV was generated by expanding 0.5 cm from the CTV in all directions, but cropped out the area with distance less than 0.3 cm from the surface.

### TVMAT and VMAT planning

**Fig. 2:**
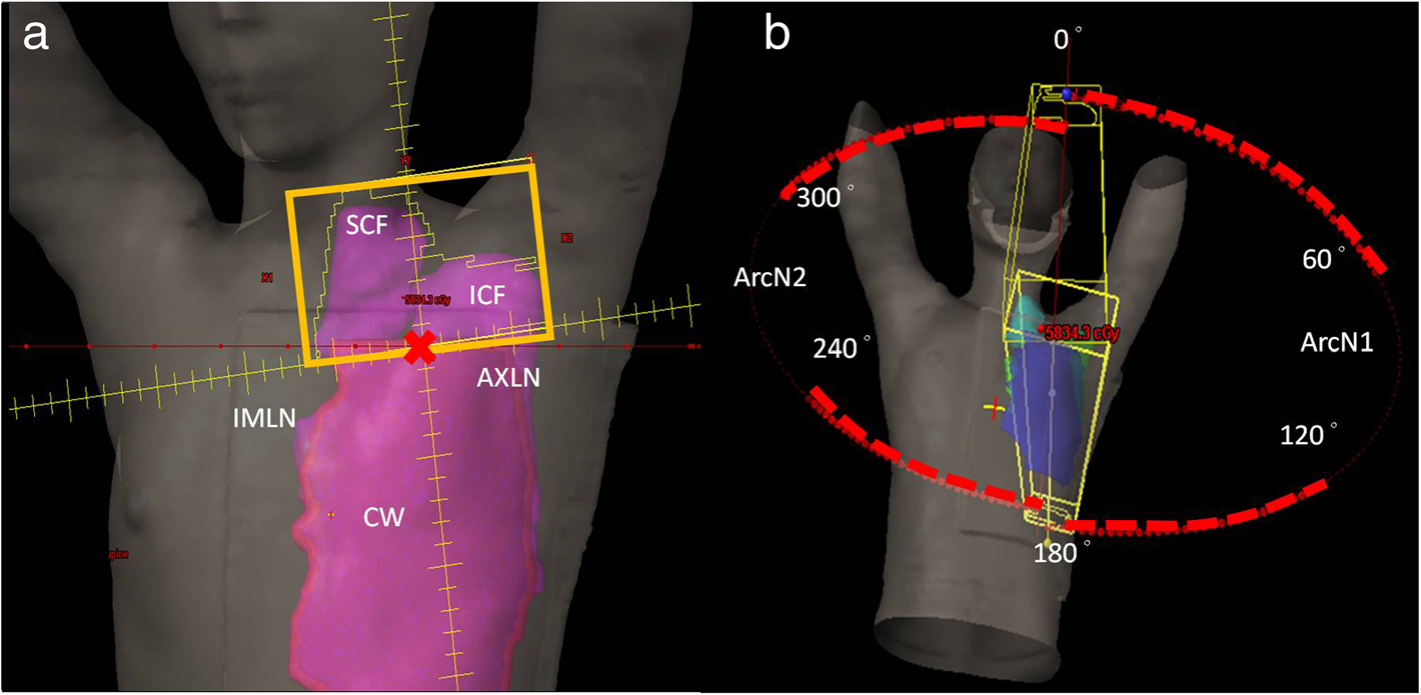
Two partial arcs (ArcN1 and ArcN2) in TVMAT were designed for SCF and ICF. Both were half-beam fields (orange rectangle in (**a**)). ArcN1 rotated from 179 to 335 degrees with an avoidance sector from 120 to 60 degrees. ArcN2 rotated from 35 to 181 degrees with an avoidance sector from 300 to 240 degrees. **a** Beam’s eye view of ArcN1. **b** Angles with radiation in ArcN1 and ArcN2 (red dash lines)

**Fig. 3:**
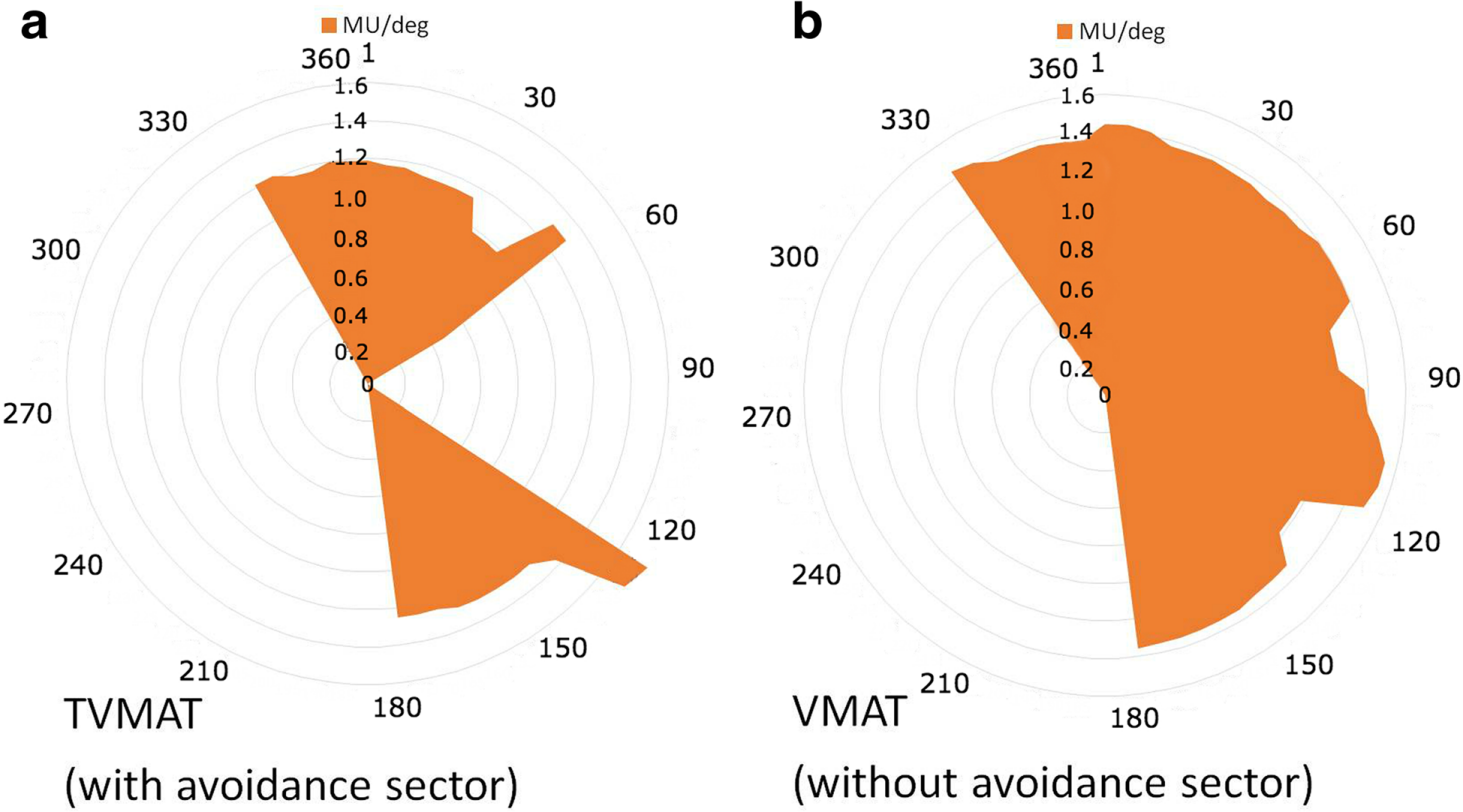
The delivery of monitor units per gantry degree (MU/deg) of ArcN1. **a** TVMAT with avoidance sector. **b** VMAT without avoidance sector

**Fig. 4:**
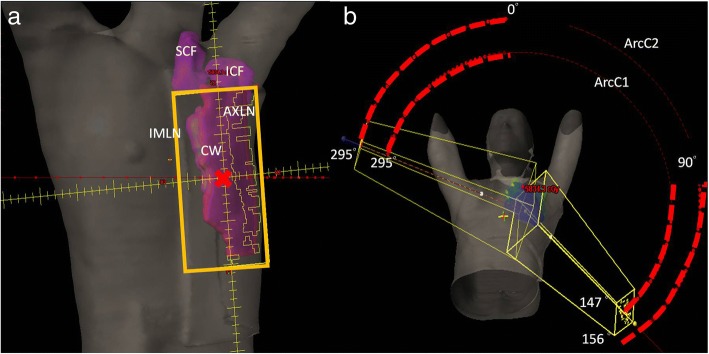
Two partial arcs (ArcC1 and ArcC2) in TVMAT were designed for CW and AXLN. ArcC1 rotated from 147 (range 130–155) to 295 (range 282–306) degrees. ArcC2 rotated from 156 (range 139–179) to 295 (range 282–306) degrees. Both had an avoidance sector from 90 to 0 degrees to reduce lung doses. **a** Beam’s eye view of ArcC1. **b** Angles with radiation in ArcC1 and ArcC2 (red dash lines)

**Fig. 5:**
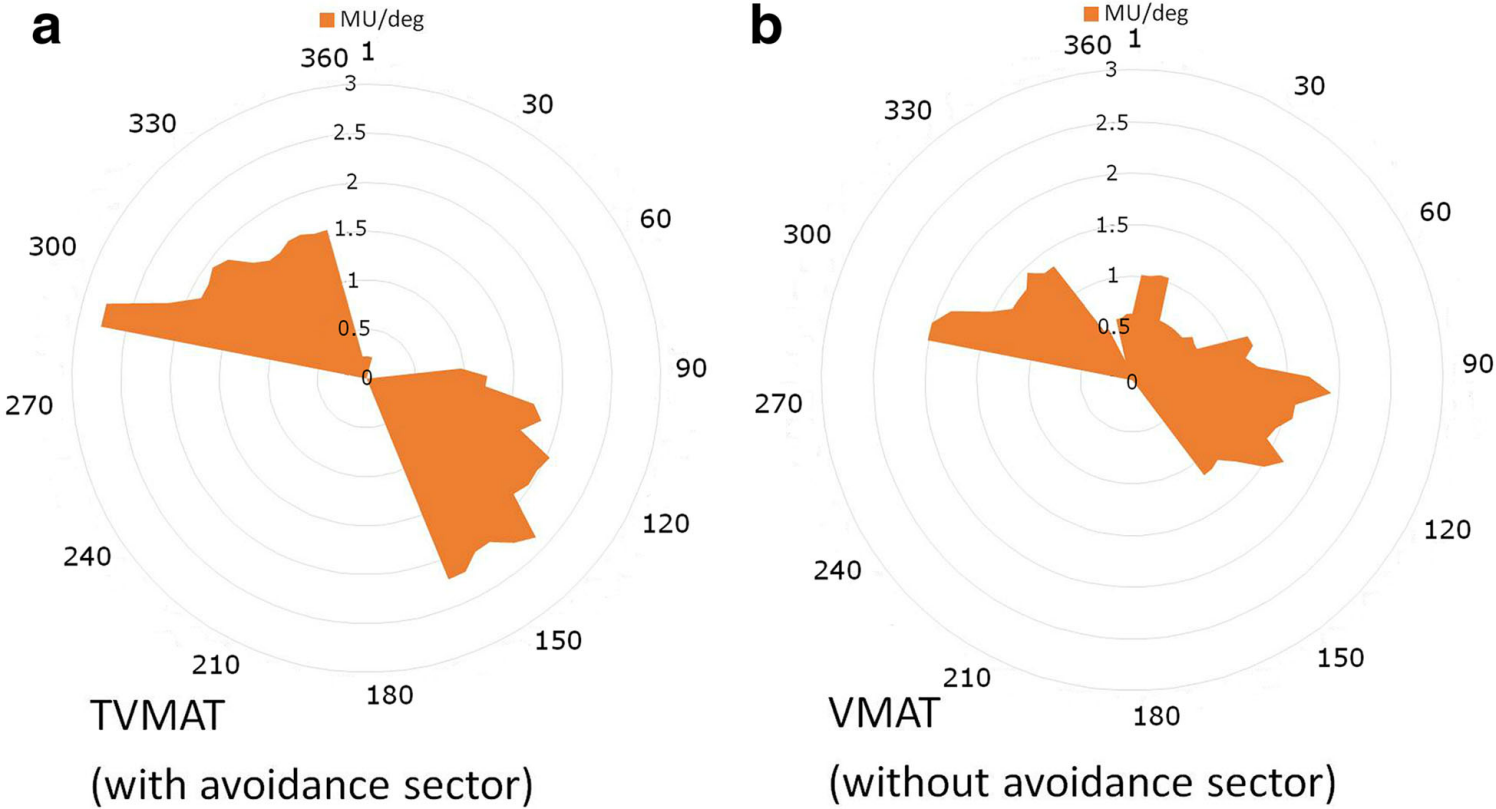
The delivery of monitor units per gantry degree (MU/deg) of ArcC1. **a** TVMAT with avoidance sector. **b** VMAT without avoidance sector

All treatment plans were designed with 5 partial arcs with 2 centers using RapidArc® (Varian Medical Systems, Palo Alto, CA, USA). In TVMAT planning, an additional avoidance sector within specific degrees of angle that had no MU delivery was used in each partial arc. Two partial arcs with half-beam fields (ArcN1 and ArcN2) were designed to irradiate SCF and ICF. ArcN1 rotated from 179 to 335 degrees with an avoidance sector from 120 to 60 degrees, and ArcN2 rotated from 35 to 181 degrees with an avoidance sector from 300 to 240 degrees (Fig. 2). Figure 3 shows the delivery of MU per gantry degree of ArcN1, which was a tangent distribution without MU delivered from the left sides of the patients in TVMAT but a semi-circle distribution in VMAT. Another 2 partial arcs (ArcC1 and ArcC2) were designed for CW and AXLN. ArcC1 rotated from 147 (range 130–155, depending on the curve of CW) to 295 (range 282–306) degrees, and ArcC2 rotated from 156 (range 139–179) to 295 (range 282–306) degrees. Both arcs had an avoidance sector from 90 to 0 degrees to reduce lung doses (Fig. 4). Figure 5 demonstrates the delivery of MU per gantry degree of ArcC1, which had no MU delivery from the left-anterior of the patients in TVMAT. ArcC3 was designed to treat IMLN. The field size was just enough to cover the volume of IMLN. It rotated from 270 (range 270–300) to 179 degrees with an avoidance sector from 60 to 120 degrees. The same degrees of partial arcs without avoidance sectors were used in VMAT planning.

The treating linear accelerator was Truebeam STx (Varian Medical Systems, Palo Alto, CA, USA), equipped with a high-definition 120-leaf multileaf collimator with 2.5 mm leaves in the middle and 5 mm leaves on the sides. The treatment plans were designed using Eclipse™ treatment planning system version 11.5 (Varian Medical Systems, Palo Alto, CA, USA). The grid size for dose calculation was 2.5 mm. The maximum dose rate was 600 MU per minute. The radiation beams were 6 MV photon beams. The Anisotropic Analytical Algorithm version 11.0.31 was used for volume dose calculation. The Progressive Resolution Optimizer version 11.0.31 was used for planning optimization.

### Planning optimization

**Fig. 6:**
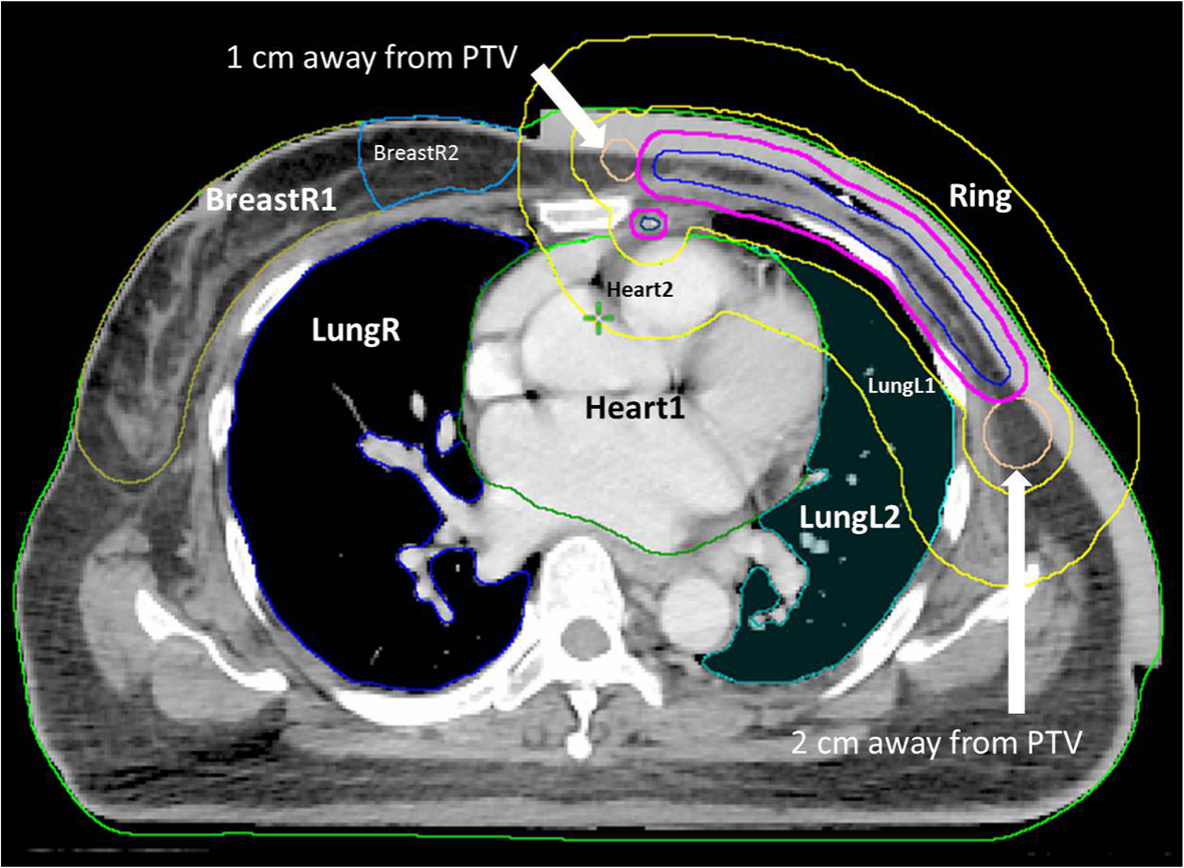
Delineation for planning optimization. All structures were at least 0.6 cm away from PTV (magenta line). A 2.0-cm wide ring structure (between two yellow lines) was designed for optimization to control the dose falloff. It was 0.6–2.6 cm away from PTV towards left lung, 1.6–3.6 cm away from PTV towards right breast, and 2.6–4.6 cm away from PTV towards back. Left lung and heart were both designed into two parts depending on whether they were within the ring structure (LungL1 and Heart2) or not (LungL2 and Heart1). The breast tissue within the 5 cm from the medial side was defined as BreastR2, and the rest was BreastR1

The prescribed dose was 50.0 Gy in 2.0 Gy per fraction. Figure 6 illustrates the delineation for planning optimization. It included a 2.0-cm wide ring structure to control the dose falloff, 1 part of the whole right lung, and 2 parts of the left lung, heart, and right breast. Bilateral shoulders were also delineated for optimization to prevent dose attenuation at the shoulders. Table 1 summarizes the objectives for planning optimization regarding each structure. The main scheme was to achieve the D98% of PTV more than 47.5 Gy, which was 95% of the prescribed dose, while maintaining the V20 of the left lung at less than 30% and keeping the mean dose to the right lung less than 5 Gy. An experienced medical physicist made all plans with minimal individual adjustments for different patients.

**Table 1:** Dose parameters and priorities for planning optimization

Structure	Volume [%]	Dose [cGy]	Priority	Structure	Volume [%]	Dose [cGy]	Priority	Structure	Volume [%]	Dose [cGy]	Priority
PTV	0	5163	950	LungL1	41	0	550	BreastR1	17	668	450
	15	5162	710		30	277	550		11	723	450
	100	5160	800		23	580	550		8	774	450
	98	5161	750		14	1228	550		3	838	450
					8	1742	550		0	870	450
					4	2281	550				
Ring	42	1697	100	LungL2	28	0	500	BreastR2	14	891	400
	31	2214	100		20	41	500		9	906	400
	19	2883	100		12	222	500		5	975	400
	10	3342	100		7	415	500		2	1050	400
	3	4080	100		1	711	500		0	1089	400
LungR	27	314	300	Heart1	43	1135	300	ShoulderL	6	843	300
	18	402	300		35	1248	300		3	1009	300
	10	625	300		28	1484	300		0	1142	300
	4	862	300		19	1801	300				
	0	1139	300		12	2209	300				
					6	2710	300				
				Heart2	23	1042	350	ShoulderR	7	552	300
					17	1171	350		3	617	300
					13	1311	350		0	756	300
					8	1465	350				
					5	1598	350				

### Plan evaluation

The CI and HI with respect to the whole PTV between TVMAT and VMAT groups were compared [10, 11]:

$$ \mathrm{CI}=\frac{{\mathrm{V}}_{95\%\mathrm{PTV}}^2}{{\mathrm{V}}_{\mathrm{PTV}}\ast \mathrm{V}95\%} $$

where V_95%PTV_ is the volume receiving at least 95% of prescribed dose in PTV, V_PTV_ is the whole PTV volume, and V95% is the whole volume receiving at least 95% of the prescribed dose.

$$ \mathrm{HI}=\frac{\mathrm{D}2\%-\mathrm{D}98\%}{\mathrm{D}50\%} $$

where D2%, D98%, and D50% are the minimum doses that the most exposed 2, 98, and 50% of PTV receive, respectively.

The D98% and D2% of PTV regarding left chest wall and lymph node regions were also assessed. Several dose parameters concerning the dose received by each OAR were evaluated, including right breast (mean dose, D2%, V10, and V5), right lung (mean dose, D2%, V10, and V5), left lung (mean dose, D2%, V20, V10, and V5), and heart (mean dose, D2%, V30, V10, and V5).

The statistical analysis of the dosimetry data was performed using independent-sample *t* test. A *P* value less than 0.05 was considered significant.

## Results

### PTV dose evaluation

The average PTV volume was 857 ± 252 cm^3^ for TVMAT and 881 ± 223 cm^3^ for VMAT, respectively. Table 2 shows the comparison of PTV doses between TVMAT and VMAT. The CI and HI of the whole PTV showed high conformity (CI: 0.98) and homogeneity (HI: 0.12) in TVMAT plans, which were compatible with VMAT plans (*P* = 0.431 and 0.177). The D98% and D2% of PTV regarding left chest wall (*P* = 0.449 and 0.747) and lymph node regions (*P* = 0.319 and 0.117) were all statistically equivalent as well. The overall results indicated that the treatment efficacies of TVMAT and VMAT were compatible.

**Table 2:** PTV dose evaluation

		TVMAT		VMAT		*P* value
		mean	SD	mean	SD	
PTV	CI	0.98	0.02	0.98	0.03	0.431
	HI	0.12	0.03	0.11	0.05	0.177
PTV-CW	D98%[Gy]	49.2	0.9	48.8	2.3	0.449
	D2%[Gy]	55.0	0.6	54.6	0.8	0.747
PTV-LN	D98%[Gy]	48.9	1.3	49.7	0.7	0.319
(SCF, ICF, AXLN, IMLN)	D2%[Gy]	55.0	0.7	54.4	0.9	0.117

Abbreviations*: TVMAT* Tangent-based volumetric modulated arc therapy, *VMAT* Volumetric modulated arc therapy, *SD* Standard deviation, *PTV* Planning target volume, *CW* Chest wall, *LN* Lymph node region, *SCF* Supraclavicular fossa, *ICF* Infraclavicular fossa, *AXLN* Axillary lymph node region, *IMLN* Internal mammary lymph node region, *CI* Conformity index, *HI* Homogeneity index

### OAR dose evaluation

**Fig. 7:**
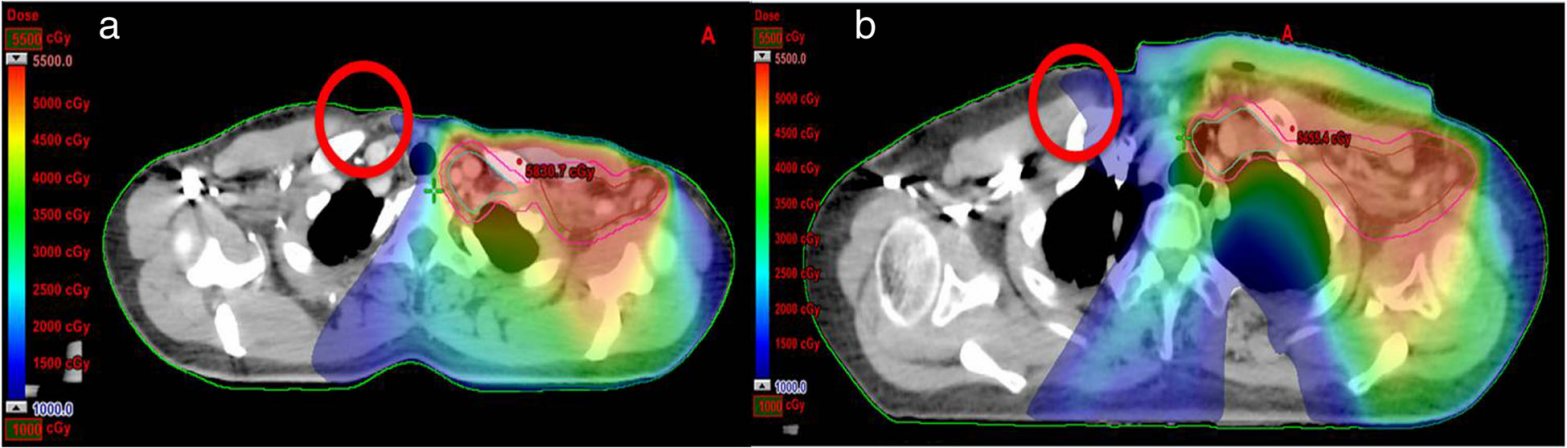
Dose distribution at the level of SCF and ICF. The red circles indicate the reduction in low dose of right breast by using **a** TVMAT compared with **b** VMAT

**Fig. 8:**
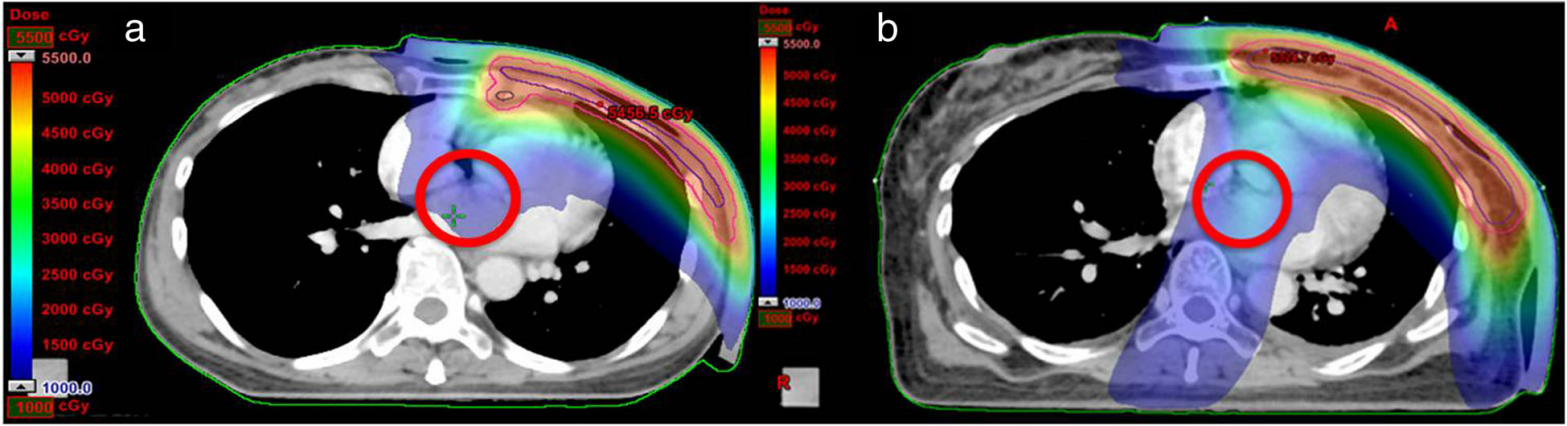
Dose distribution at the level of CW. The red circles indicate the reduction in low dose of heart by using **a** TVMAT compared with **b** VMAT

Table 3 demonstrates the dose reductions of OAR in TVMAT compared with VMAT, which were especially large for the contralateral organs (right breast and right lung) and under low dose parameters (V10 and V5). The right lung dose at V10 in TVMAT accounted for the greatest reduction by a percentage regarding the assessed parameters of all organs and was only 13.7% of that in VMAT. The right breast also showed significantly lower doses of all assessed parameters in TVMAT. Figure 7 illustrates the area of low dose reduction of the right breast. Moreover, the mean heart doses of every patient in TVMAT were lower than 15 Gy and at least 3.2 Gy less than that in VMAT. Otherwise, the dose at V5 was significantly reduced in TVMAT. Figure 8 illustrates the area where the heart had lower doses. The percentages of dose reduction in the left lung were not so tremendous, but still had statistical significance at V20, V10, and V5.

**Table 3:** OAR dose evaluation

		TVMAT		VMAT		*P* value
		mean	SD	mean	SD	
Right breast	mean dose [Gy]	3.7 (46.8%)	1.4	7.9	3.0	0.001*
	D2% [Gy]	12.9 (63.5%)	4.8	20.3	7.4	0.029*
	V10 [%]	7.4 (27.6%)	7.4	26.8	21.4	0.011*
	V5 [%]	25.9 (43.6%)	13.5	59.4	17.1	< 0.001*
Right lung	mean dose [Gy]	3.6 (63.2%)	1.1	5.7	1.3	< 0.001*
	D2% [Gy]	10.4 (67.1%)	2.2	15.5	4.9	0.004*
	V10 [%]	2.9 (13.7%)	2.3	21.1	16.5	0.003*
	V5 [%]	23.9 (71.6%)	12.2	33.4	22.4	0.431
Left lung	mean dose [Gy]	15.5 (91.2%)	1.4	17.0	1.1	0.052
	D2% [Gy]	50.2 (101.0%)	1.1	49.7	1.9	0.354
	V20 [%]	27.3 (89.5%)	2.4	30.5	2.2	0.013*
	V10 [%]	45.7 (87.5%)	5.1	52.2	5.5	0.030*
	V5 [%]	74.6 (87.4%)	11.5	85.4	5.0	0.016*
Heart	mean dose [Gy]	11.7 (78.5%)	3.0	14.9	4.5	0.062
	D2% [Gy]	40.4 (99.5%)	8.3	40.6	9.1	1.000
	V30 [%]	7.2 (69.2%)	4.0	10.4	9.7	0.708
	V10 [%]	41.7 (74.9%)	16.3	55.7	17.8	0.062
	V5 [%]	75.7 (81.7%)	15.4	92.6	10.1	0.030*

*Statistical significance

Abbreviations*: TVMAT* Tangent-based volumetric modulated arc therapy, *VMAT* Volumetric modulated arc therapy, *SD* Standard deviation

### Plan objectives

The average treatment time of TVMAT was 2.3 ± 0.4 min with an average of 973 ± 189.6 MU being delivered, which was less than VMAT with 1305.5 ± 137.6 MU being delivered in 3.8 ± 0.9 min.

## Discussion

VMAT is generally associated with a larger volume of low dose spreading to healthy tissues, which leads to concerns about increasing the risk of secondary malignancies. Although the actual doses received by the contralateral breast and lung are low, the probabilities of secondary cancers are notable. The value of excess relative risk per Gy for secondary cancer in the contralateral breast following radiotherapy of breast cancer is 0.86 Gy^− 1^, which is reduced by a factor of 2 and set to 0.43 Gy^− 1^ in fractionated radiotherapy [12–14]. For patients diagnosed with a second primary lung cancer 5 or more years after breast cancer treatment, the rate of lung cancer increases linearly with 8.5% per Gy. This rate is even enhanced for ever-smokers with an excess rate of 17.3% per Gy [15]. However, it is difficult to abandon VMAT because it generally has a better performance at intermediate-high dose sparing and mean heart dose, which is related to ischemic heart disease [6–9].

In the present study, TVMAT comprehensively improved the disadvantages of VMAT. The analysis demonstrated a statistically significant reduction in almost all low dose parameters and contralateral organ parameters. The only contralateral organ parameter that had a reduced but insignificant result was V5 of the right lung. It is probably due to the relatively large standard deviations, which are related to fewer doses delivered to the right lung, and hence large variations between patients. However, in fact, the average V5 of the right lung was reduced from 33.4% in VMAT to 23.9% in TVMAT. Using TVMAT may relieve the worries about secondary cancers while preserving the advantages of VMAT.

Some earlier studies used tangential arcs or avoidance sectors in order to improve the dose distribution of VMAT for treating left breast cancer. Fogliata et al. applied an avoidance sector from 0 to 105 degrees to do whole breast irradiation, which presented a reduction of the mean doses for all critical structures with trade-offs of high-dose spillage in the healthy tissue and higher skin dose [16]. Pasler et al. conducted a technique with 2 small tangential arc segments less than 60 degrees and compared it to large-angle VMAT more than 230 degrees for patients needing left breast, supraclavicular lymph nodes, and infraclavicular lymph nodes irradiation. They concluded that the technique provided considerably reduced low dose, especially in the heart and contralateral structures, with the cost of a slightly inferior target coverage and homogeneity [17]. Kuo et al. introduced 5 partial arcs with 3 from the anterior and 2 from the side for the patients with expander or implant reconstructions. The modified VMAT technique avoiding the ipsilateral arm can produce acceptable clinical plans regarding post-mastectomy radiotherapy [18].

The application of 2 centers in TVMAT is unique and noteworthy. Because the anatomy geometries at the upper level over SCF and ICF and the lower level over CW and AXLN are different, it is warranted to separate these 2 parts in treatment planning. The beam angles are essential for taking care of both target coverage and OAR sparing. To make a rainbow-shaped isodose curve over CW and AXLN, the tangent-based beams from the right-anterior and left-posterior of the patients are essential for prevention of the beam penetrating through the body, which is quite different to the beams arranged for SCF and ICF in the anterior-posterior direction to avoid dose to the contralateral breast. Therefore, 2 centers were used in TVMAT other than the feature of avoidance sectors in specific degrees. Furthermore, for a better dose control at the junction of 2 fields, ArcN1 and ArcN2 were set up in half-beam fields refraining beam diversion.

IMLN irradiation is strongly considered for nodal positive patients [2–4]. Although sampling of internal mammary sentinel nodes is not performed routinely in contemporary practice, it has to be mentioned that the first filter stations for the lymphatic drainage of breasts are AXLN and IMLN [19]. The incidence of IMLN metastasis is highly correlated with tumor location and AXLN status, which is only 4.4% with no AXLN metastasis, but increases to 18.8% with 1–3 AXLN metastases, 28.1% with 4–6 AXLN metastases, and 41.5% with ≧7 AXLN metastases [20]. In order to treat IMLN, which may generally increase ipsilateral lung dose, DIBH with wide tangents or 3-dimensional conformal radiotherapy (3DCRT) was most employed [21–23]. Osman et al. further compared VMAT with 3DCRT in patients with or without DIBH. They discovered the dose benefits in a better target coverage and a reduction of ipsilateral lung dose and cumulative heart dose with VMAT in DIBH, but at the expense of increasing the dose to the contralateral breast [24].

We used ArcC3 to solve the problem caused by IMLN irradiation. It rendered a field just wide enough to cover the volume of IMLN with specific rotation degrees designed for IMLN. Concerning IMLN locating at the anterior and leaning to the left of the body, the beam contributed more from the anterior than posterior, not directly from the anterior-posterior direction but slightly from the right-anterior to the left-posterior. As the beam angles are essential for taking care of both target coverage and OAR sparing, we took them into account for every individual target towards a more specific approach.

This study has some limitations. First, the CT simulation was performed under free-breathing conditions but not in a DIBH setting. The concern was the feasibility of DIBH with VMAT-based planning in clinical implementation. To perform a successful treatment with DIBH, patients have to keep their breath at a consistent and stable level, not only during CT simulation but throughout the whole treatment process. The average treatment time of TVMAT and VMAT was 2.3 and 3.8 min in the study, respectively, which is much longer than people can hold their breath. Therefore, a 4-dimensional CT-based planning and respiratory gating treatment system is mandatory to assist in DIBH treatment with VMAT-based planning. In an institution with cooperative patients and adequate equipment, DIBH with TVMAT planning may further reduce OAR doses compared with VMAT planning. Second, although 2 centers were essential in TVMAT for different beam angles regarding anatomy geometries and half-beam fields were used to avoid beam diversion, setup errors, particularly in the longitudinal axis, may have resulted in dose deviations. In our assessment, hot or cold spots at the junction with about 30% dose deviations presented in recalculation by shifting a center by 0.5 cm in the longitudinal axis after planning. In clinical application, this warrants combining 2 centers to make a single plan, recording the distance between the 2 centers, and moving a patient exactly according to the plan using an automated moving couch with quality assurance. Finally, it is necessary to pay attention to the bolus used in the study. A thick and wide enough bolus, 1 cm in thickness and covering the whole left chest wall, was applied to each patient. One benefit of the bolus was to achieve our prescribed skin dose, which was 50.0 Gy in the study, because skin involvement is a risk in advanced breast cancer. The other benefit was to solve the problem of breathing-induced shifts of the chest wall. The shifts were considered in the target delineation because CTV can directly expand into the bolus on the image to obtain PTV. Because it is not easy to open up multileaf collimators in VMAT-based planning and because TVMAT forces the radiation beams to be mainly tangential, such respiratory organ motions have to be taken into account beforehand in target delineation. For the patients without a bolus, we recommend expanding the contour into the air and designing TVMAT with a replaced virtual bolus in planning. The above considerations are the necessary precautions when employing TVMAT in clinical practice. TVMAT can be applied properly with attention to the details.

## Conclusion

TVMAT is an innovative radiotherapy planning technique designed for advanced left breast cancer. It comprehensively improves the disadvantages of traditional VMAT with respect to low dose spreading and detriments of contralateral organs on treatment plan dosimetry while maintaining target coverage and homogeneity. It creates a paradigm for management of difficult cases needing nodal irradiation in clinical applications.

## Abbreviations

3DCRT: 3-dimensional conformal radiotherapy
AXLN: Axillary lymph node region
CI: Conformity index
CT: Computed tomography
CTV: Clinical target volume
CW: Chest wall
DIBH: Deep inspiration breath-hold
Gy: Gray
HI: Homogeneity index
ICF: Infraclavicular fossa
IMLN: Internal mammary lymph node region
IMRT: Intensity-modulated radiation therapy
MRM: Modified radical mastectomy
MU: Monitor unit
OAR: Organs at risk
PTV: Planning target volume
SCF: Supraclavicular fossa
TVMAT: Tangent-based volumetric modulated arc therapy
VMAT: Volumetric modulated arc therapy
